# Efficient functional cyst formation of biliary epithelial cells using microwells for potential bile duct organisation *in vitro*

**DOI:** 10.1038/s41598-018-29464-w

**Published:** 2018-07-23

**Authors:** Astia Rizki-Safitri, Marie Shinohara, Yasushi Miura, Mathieu Danoy, Minoru Tanaka, Atsushi Miyajima, Yasuyuki Sakai

**Affiliations:** 1 Center for International Research on Integrative Biomedical Systems (CIBiS), Institute of Industrial Science (IIS), The University of Tokyo, Tokyo, Japan; 2 Department of Life Science and Medical Bio-Science, School of Advanced Science and Engineering, Waseda University, Tokyo, Japan; 3 LIMMS/CNRS UMI2820, Institute of Industrial Science (IIS), The University of Tokyo, Tokyo, Japan; 4 Institut d’Electronique, de Microélectronique et de Nanotechnologies (IEMN), Université Lille, Lille, France; 5 Laboratory of Cell Growth and Differentiation, Institute of Molecular and Cellular Bioscience, The University of Tokyo, Tokyo, Japan; 6 National center for global medicine (NCGM), Tokyo, Japan; 7 Department of Chemical System Engineering, Graduate School of Engineering, The University of Tokyo, Tokyo, Japan; 8 Max Planck-The University of Tokyo, Center for Integrative Inflammology, The University of Tokyo, Tokyo, Japan

## Abstract

Establishing a bile duct *in vitro* is valuable to obtain relevant hepatic tissue culture systems for cell-based assays in chemical and drug metabolism analyses. The cyst constitutes the initial morphogenesis for bile duct formation from biliary epithelial cells (BECs) and serves the main building block of bile duct network morphogenesis from the ductal plate during embryogenesis in rodents. Cysts have been commonly cultured via Matrigel-embedded culture, which does not allow structural organisation and restricts the productivity and homogeneity of cysts. In this study, we propose a new method utilising oxygen permeable honeycomb microwells for efficient cyst establishment. Primary mouse BECs were seeded on four sizes of honeycomb microwell (46, 76, 126, and 326 µm-size in diameter). Matrigel in various concentrations was added to assist in cyst formation. The dimension accommodated by microwells was shown to play an important role in effective cyst formation. Cytological morphology, bile acid transportation, and gene expression of the cysts confirmed the favourable basic bile duct function compared to that obtained using Matrigel-embedded culture. Our method is expected to contribute to engineered *in vitro* liver tissue formation for cell-based assays.

## Introduction

The bile duct is configured by biliary epithelial cells (BECs; also known as cholangiocytes) and comprises a finely organised biliary network. It is responsible for bile acid collection and transportation from the bile canaliculi among hepatocytes in the *in vivo* hepatic system^1^. The absence of BECs in either monolayer^2–4^ or three-dimensional (3D) hepatocyte culture *in vitro*^5^ prevents the reconstruction of relevant hepatic tissue. Alternatively, a 3D spheroid co-culture system using hepatocytes and BECs has been shown to demonstrate possibility to mimic liver-related function *in vitro*^6,7^. Nevertheless, a proper collection of bile acids remain also, highly desirable in the *in vitro* cell-based hepatotoxicity assays.

To date, the limited research in this field has yet been unable to establish a functional culture for bile ducts. One study demonstrated that rat BECs in a 3D collagen sandwich culture in the presence of dimethylsulphoxide express both the morphology and the functional activities of ductular ultrastructures^8^. However, the development of this structure was time-consuming (44 days) and the constructed bile duct architecture was discontinuous and it is not suitable for bile acid collection. Another cellular aggregate-based study employing primary rat BECs and foetal rat hepatocytes reported the occurrence of bile acid drainage towards the arbitrarily-formed bile poles owing to the role of polarised-segmented bile duct network in the aggregates^6^. Therefore, a relevant bile duct structure for appropriate bile acid drainage and recovery is highly desirable.

Another approach is to develop a cyst that is characterised as a spheroid sac shape with a central lumen and comprised of a number of BECs^8–10^. In particular, the geometric construction model of dynamic 3D morphogenesis of the mouse bile duct network explicated that the cyst-structures are generated from the ductal plate^11–13^, which could be regarded as a building block comprised of a long luminal structure along the foetal hepatic portal vein during mouse embryogenesis^14,15^. Corresponding to the *in vivo* condition, a cyst could also be established by BECs under a 3D extracellular matrix (ECM)-based culture microenvironment^16–18^, with features that are uniquely distinguished from those of other liver cells^19,20^. Such cysts were also able to emphasise the functional characteristic of BECs as related to the bile efflux inwards and outwards from the lumen^8,9,17,18^ in the laminin-rich ECM^9,17^. Notably, this characteristic is specifically utilized as the main indicator to differentiate BECs from induced pluripotent stem cells (iPSCs)^8,18,19^. However, the prevalent *in vitro* experiments of cyst establishment using conventional Matrigel-embedded culture^8,9,18,19^ face various drawbacks such as inconsistent cyst formation and the lack of robust method of cyst harvesting for subsequent studies.

In comparison, honeycomb-shaped microwells fabricated from poly(dimethylsiloxane) (PDMS) are largely used for cell morphology and behaviour control, particularly for aggregation-based studies^20,21^. The PDMS material permits direct oxygenation throughout the culture system, causing the development of appropriately thick layers of hepatic tissue culture^3^ and large inoculum density per unit area^19,22^. The association between the PDMS-honeycomb microwell and PDMS-bottom culture plate provides oxygen supplies 80 times higher than those in a polystyrene plate, which markedly enhances the cell productivity per unit area^20^. Hence, the oxygen-permeable microwell is feasibly suited as an alternative method for efficient size-regulated cyst formation.

In consideration of such factors, we proposed an efficient method to generate cysts from a primary culture of mouse BECs utilising the PDMS-honeycomb microwell. We cultured primary BECs in various honeycomb microwell-sizes and Matrigel supplementation. The microwell was expected to provide strict size control of the cysts, resulting in their homogeneity and enabling the bile acid collection as well as convenient harvesting method for further analyses, thereby overcoming the limitation of Matrigel-embedded culture. We considered that the cyst may represent a favourable recourse for bile acid collection and the establishment of derived-bile duct structures *in vitro*. In addition, the finding from this study may lead to the establishment of *in vitro* engineered bile duct networks *in vitro* from stable cysts, mimicking the networking of bile duct observed embryonic morphogenesis.

## Results

### BEC cysts either develop from single cells or small aggregation

**Figure 1:**
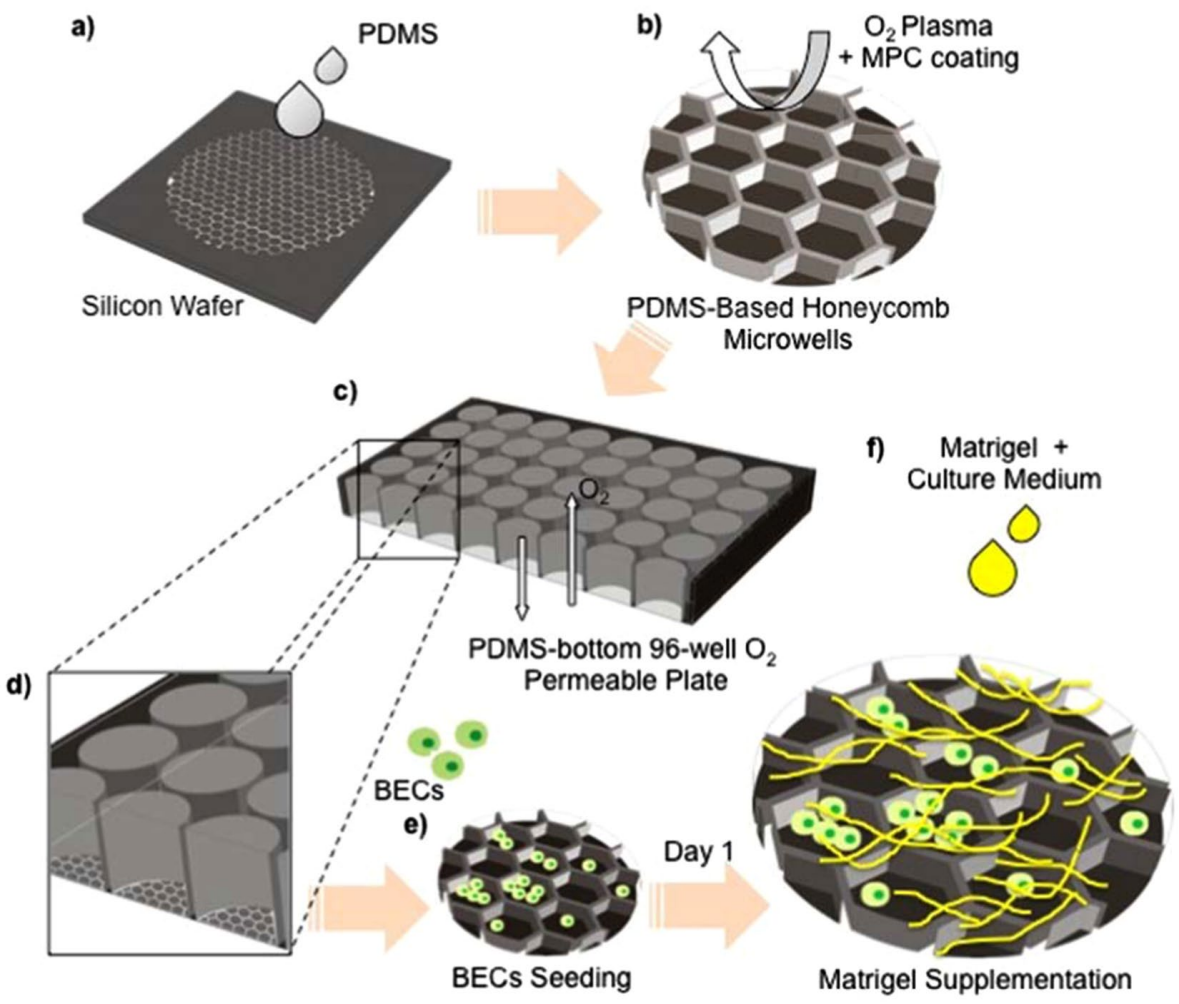
Fabrication of PDMS-based honeycomb microwells followed by the BEC seeding protocol. PDMS-based honeycomb microwells were generated by employing a silicon wafer replica-mould (**a**) and treated by MPC coating (**b**) prior to insertion on the PDMS-bottom 96-well oxygen (O_2_) permeable plate (**c**,**d**). The O_2_ permeable-plate provides the direct oxygenation and prolongs the culture duration as well as mimicking the actual *in vivo* condition. Matrigel supplementation was added one day after inoculation in order to prevent cell adherence (**e**,**f**).

**Figure 2:**
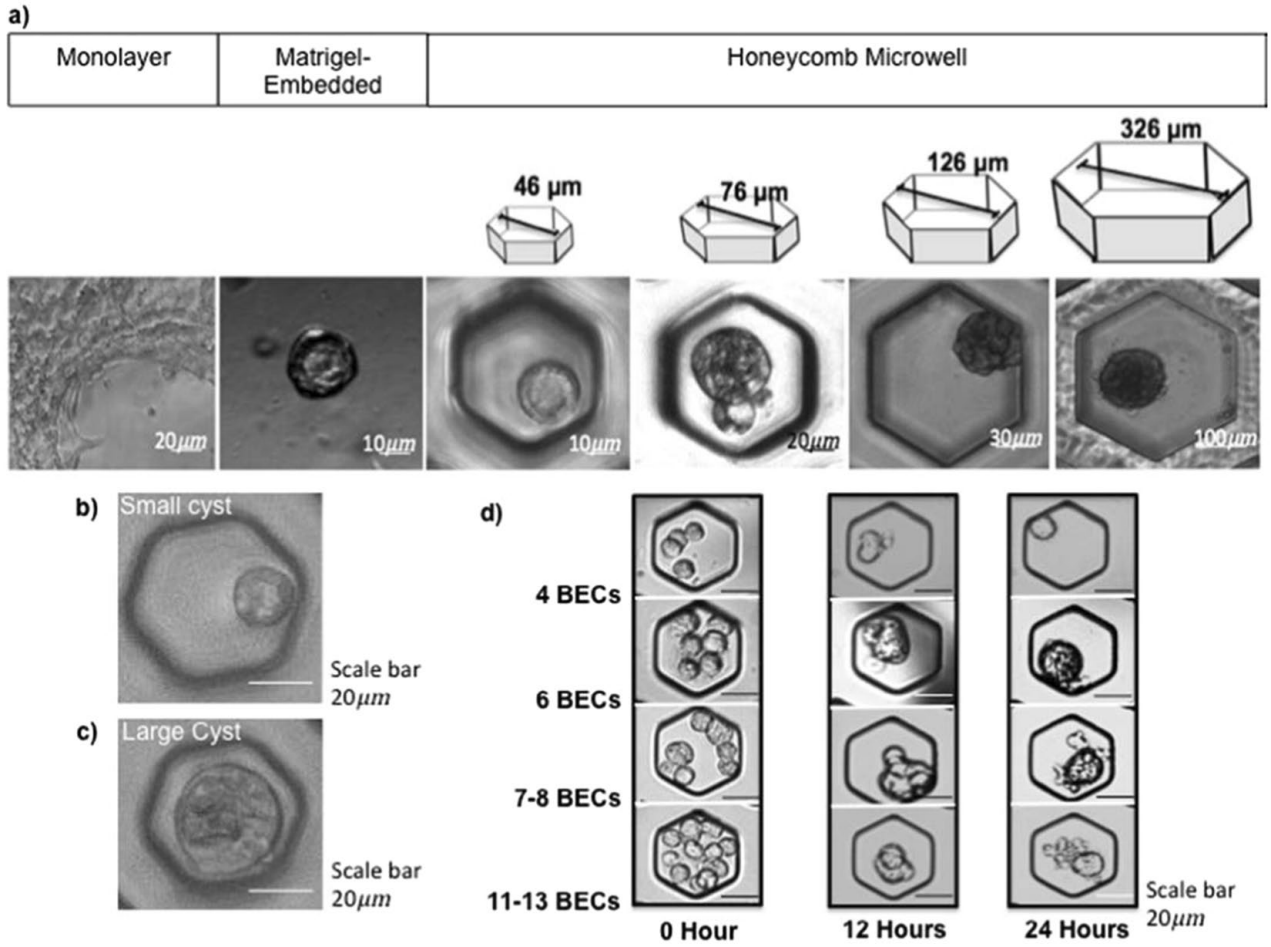
Cyst establishment in various honeycomb microwell sizes compared to in Matrigel-embedded culture. After day 3, cysts in 46- and 76-µm-size microwells resembled the morphology corresponding to the cysts in the Matrigel culture, despite the absence of Matrigel addition at day 1 culture after seeding. Optimum seeding densities for each well-size were employed for these cultures (**a**). Larger sizes of honeycomb were unable to support cyst formation and the cells remained as aggregates. Aggregates assembled from up to six BECs developed into cysts whereas cyst formation was aborted in larger aggregates. The morphology of the cyst originating from a single cell (**b**) and cell aggregations (**c**) in 46-µm-size microwells at day 3 of culture. The cyst formation following BEC aggregation was recorded within 24 h in the 76-µm-size microwell (**d**).

The cyst initially developed within three days after the seeding process of the mouse primary BECs (Fig. 1a–f). Both the Matrigel-embedded culture and any size of 2-methacryloyloxiethyl phosphorylcholine (MPC)-coated honeycomb microwell allowed BEC aggregation. MPC coating was conducted to prevent cells adhesion^23,24^. We also determined the optimum seeding density for each microwell size (Table 1). The optimum densities were decided according to the highest probability of the cysts to be observed per image (4 images, n = 4 from 2 independent experiments). Using the optimum seeding densities, cysts were mostly observed in the 46- and 76-µm-size honeycomb microwells after day 3 of culture (Fig. 2a). The 46-µm-size was preferably used to assist cyst formation as it exhibited optimal size-controlled maintenance of BEC aggregation in comparison to that of the other sizes. BEC aggregation in the bigger honeycomb microwell sizes (126 and 326 µm) also occurred albeit without subsequent cyst formation. Cysts were more likely to develop from a single cell (Fig. 2b) or a small aggregation of BECs, up to six cells (4.1 ± 1.64 cells per 46-µm-size well) (Fig. 2c). This situation also explained the observation of some cysts in 76-µm and larger microwells (i.e., 126- and 326-µm-size), as long as the microwell BEC congregates consisted of not more than six cells. Conversely, we observed large aggregates that consisted of smaller cysts closely packed together in the 126- and 326-µm-size microwells. In comparison, the origin of the BEC cysts was unable to be determined in the Matrigel-embedded culture as a clear mechanism of cyst-formation could not be elucidated.

**Table 1:** Cysts were qualitatively observed in various seeding densities and honeycomb microwell diameter sizes after 3 days of cultures.

Seeding Density (per cm^2^)	Diameter Size of the Honeycomb Microwell (μm)			
	46	76	126	326
1 × 10^4^	+	+		
2 × 10^4^	+	+		
4 × 10^4^	++	++	+	−
6 × 10^4^	+++	+++	+	−
8 × 10^4^	++	+++	−	−
1 × 10^5^	−	+	−	−
2 × 10^5^	−	−	−	−

^−^No cyst, only aggregates.

^+^A few cysts.

^++^Up to half are cysts.

^+++^More than half are cysts.

Grey columns: no experiment was conducted using these densities.

To clarify whether the aggregation may lead to cyst formation, real-time recording was conducted to reveal the origin of the cyst itself. We used a 76-µm-size honeycomb microwell for the recording as it provided wide varieties of the number of BECs clustered in each well, ranging from 0–15 cells/well (10.1 ± 3.11). The recorded BEC aggregation was relatively rapid, occurring within only 12 h and followed by cyst formation up to 24 h in total (Fig. 2d). Moreover, aggregations assembled from more than six cells in each well did not entirely correspond to the cyst but merely tended to configure into aggregates.

### Matrigel supplementation supports the formation and stability of the cyst in the culture system

Considering the important role of the ECM in cyst formation, BEC cultures were supplemented with Matrigel to facilitate the cyst formation. Matrigel was dissolved in the culture medium to create low viscosity Matrigel that was capable of facilitating cyst formation and also enabled convenient cyst collection without any enzymatic process. Matrigel supplementations were conducted 1 day after inoculation to prevent the dissociation of protein molecules from the matrix, which effected adherence on the MPC coating and promoted subsequent cells adhesion.

Upon applying the optimum seeding density for 46-µm-size microwells (6 × 10^4^ cells/cm^2^), the resultant cysts persisted up to 13 days in the non-Matrigel supplemented culture medium but even longer in the supplemented medium, particularly in the 150, 300, and 600 µg/ml concentration (Fig. S1).

**Figure 3:**
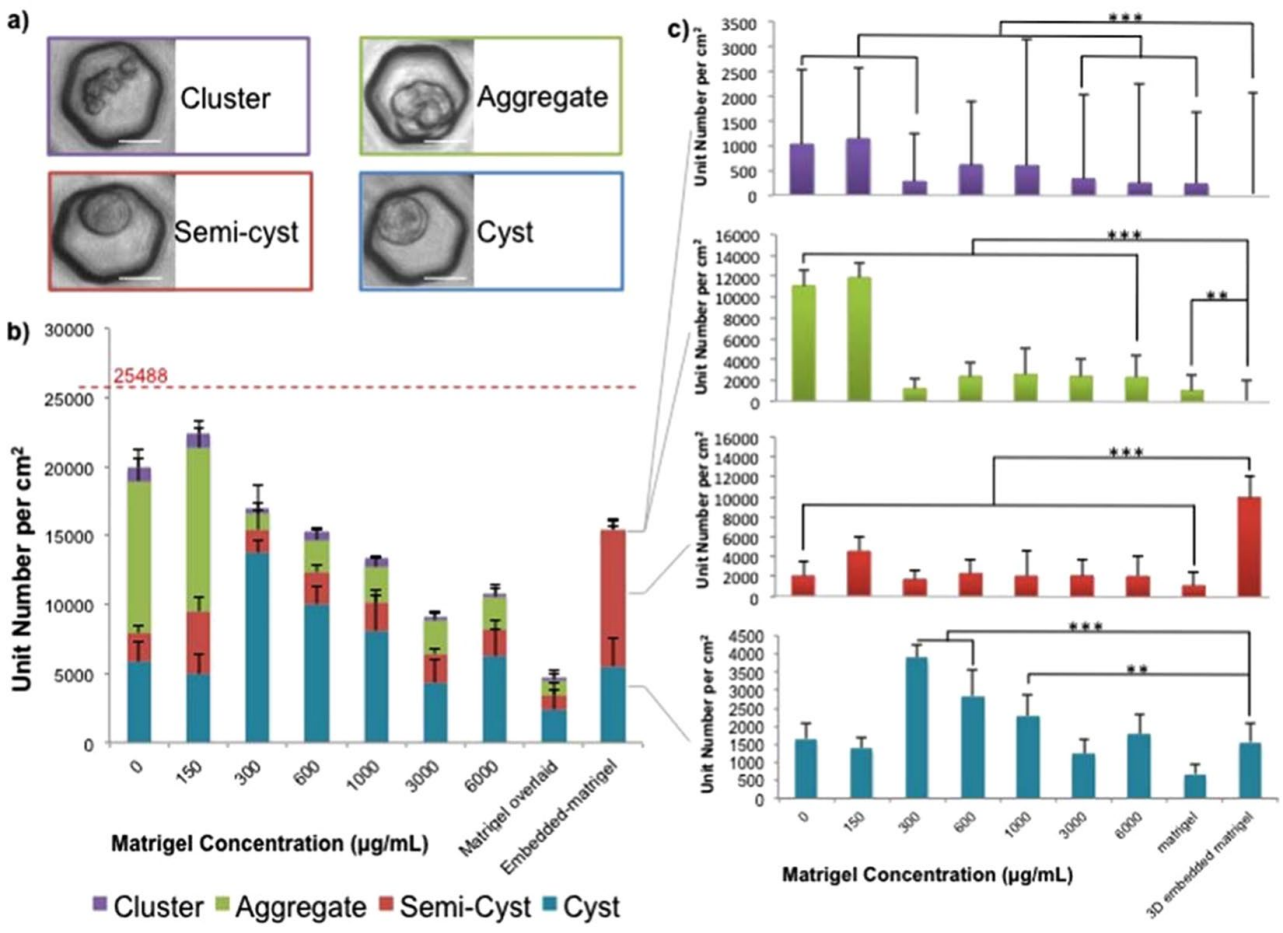
Average numbers of four types of structures per well in the honeycomb microwell in various concentration of Matrigel supplementation (per µg/ml) in day 3 culture. Four structures were observed: cluster, aggregate, semi-cyst, and cyst-structure (scale bar: 20 µm) (**a**). Total unit structures exhibited the highest numbers in Matrigel supplemented up to 150 µg/ml and gradually decreased as the supplementation increased (**b**). Red-dashed line represents the total number of microwells per cm^2^; the distance between the line and the bars shows the amount of empty wells. Cyst-structures were significantly observed in 300 to 1000 µg/ml concentration, whereas semi-cysts existed throughout Matrigel-embedded culture and aggregates were abundant in concentrations less than 300 µg/ml, compared to in Matrigel-embedded culture (**c**). Data were collected from 30 images from 3 independent experiments (n = 3) with standard deviation (*p < 0.05; **p < 0.01; ***p < 0.001).

To clearly confirm the ideal concentration of Matrigel supplementation, cysts were quantitatively counted after being cultured for 3 days. Notably, we observed four typical unit structures in the culture system: (a) cluster, characterised by a group of BECs inside the microwell without forming an aggregate; (b) aggregate; (c) semi-cyst, featured by a round-like cyst shape but lacking lumen at the apical side; and (d) cyst (Fig. 3a). In the 0 and 150 µg/ml concentration, aggregates constituted more than a half of the unit population inside the microwell. The 300 concentration appeared to be the preferable condition as indicated by the high number of both cysts and semi-cysts established, followed by 600 and 1000 µg/ml concentrations (Fig. 3b). This number was nearly equal to the number of cyst and semi-cysts developed in conventional Matrigel-embedded culture. However, the majority of the structures developed in the Matrigel-embedded culture were semi-cysts (Fig. 3c). A gelling process was revealed to have occurred in the 1000 µg/ml and higher concentration. This state captured most of the unit structures from the microwell towards the matrix and constructed bigger aggregates that also decreased the number of unit structured counted, but increased the total number of empty wells observed in the culture system. An identical experiment was also performed using the 76-µm-size honeycomb microwell applying 1 × 10^5^ cells/cm^2^ seeding density at the 300 µg/ml Matrigel concentrations. The results demonstrated that cyst production in the 46-µm-size microwell was still highly significant owing to the larger number of wells per area compared to the 76-µm-size (Fig. S2).

### Honeycomb microwell enhances the establishment of cysts in conjunction with Matrigel supplementation, both morphologically and functionally

**Figure 4:**
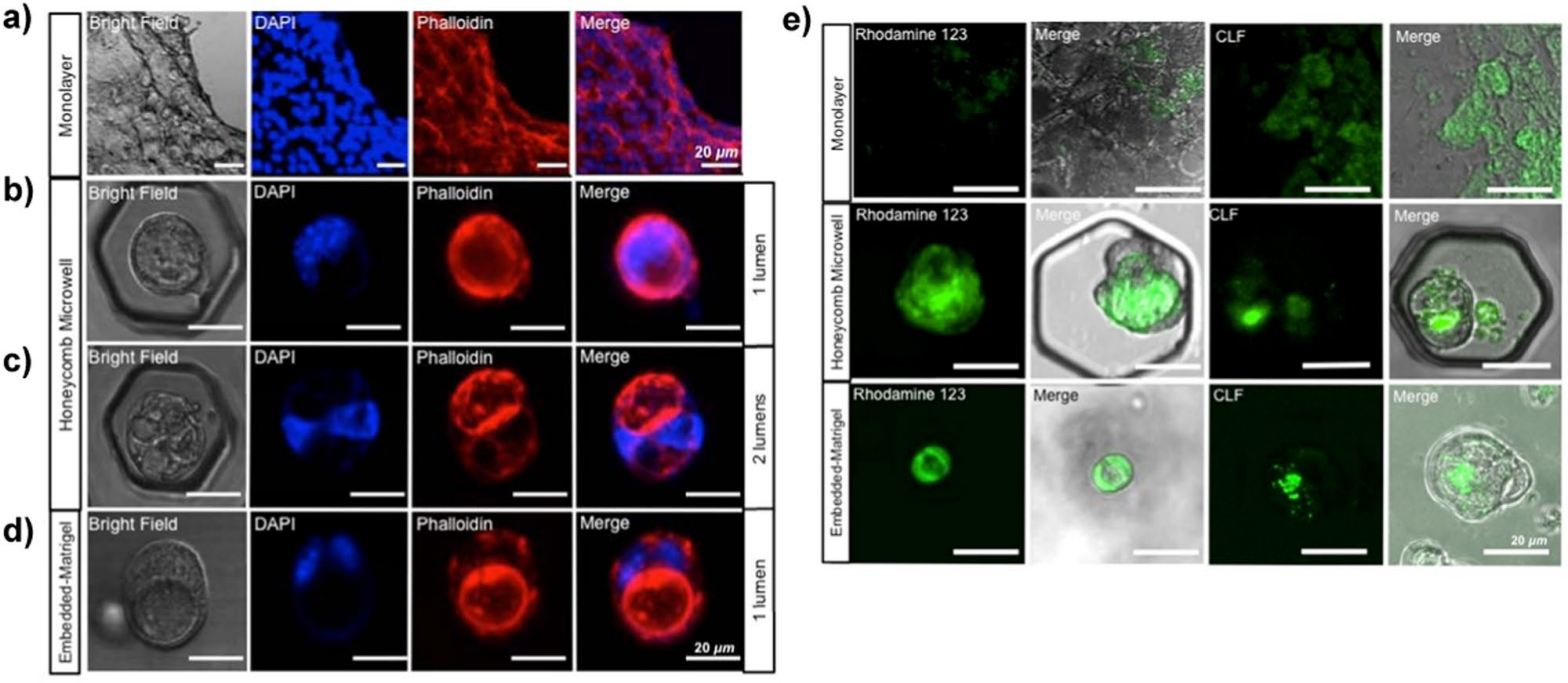
Fluorescence imaging validates the cytomorphology and the function of the cysts. Cytomorphology of the cyst evaluated using actin configuration (phalloidin) and nuclear (DAPI) staining conveyed the sac morphology (bright field). Monolayer culture lacked 3D-assembly capability owing to the cell adherence (**a**). Cysts in the honeycomb microwell were observed either in a single lumen (**b**) or 2 lumens (**c**), whereas mostly single lumens were detected in Matrigel-embedded culture (**d**) Applying an identical incubation period, cysts developed in the honeycomb microwell preferentially accumulated CLF and rhodamine 123 in the lumen compared to in cysts from embedded-Matrigel culture. The rhodamine 123 was enclosed within the cells of the cyst in Matrigel-embedded culture (**e**) (scale bar: 20 µm).

The actin structure (phalloidin) reconstructed in the cyst showed the assembly of a sac configuration of the cytoplasm, engaging the lumen structure at the apical site (Fig. 4a–d). Cysts developed both in the honeycomb microwell and the conventional Matrigel-embedded culture exhibited non-significant differences of the morphology. Notably, some of the cysts observed in microwell culture possessed more than one lumen (Fig. 4c) and those in Matrigel-embedded culture mostly had a single lumen (Fig. 4d), whereas monolayer culture did not show the sac configuration owing to the cell attachment (Fig. 4a). Multiple lumens established by the cysts in the honeycomb microwell were ascribed to the aggregation of the BECs. Thus, the aggregates were able to conveniently change their conformation.

Functional bile ducts structures require the ability to accumulate the bile acid in their lumen. Cholyllysyl fluorescence (CLF) constitutes an analogue of bile acid that is commonly utilised for the bile acid efflux-related qualitative studies such as judging transporter performance (e.g., drug or lipid transporter)^3,5,25^. In addition, rhodamine 123 fluorescein reveals a high function of multidrug resistance protein 1 (MDR1), which is related to apical trans-membrane efflux pump activity towards lipophilic cytotoxic drugs^8,9,20^. Cysts developed in the honeycomb microwell had functional transporters such as drug transporters and the secretin receptor, as demonstrated by CLF accumulation. These cysts also accumulated rhodamine 123 dye vividly as opposed to cysts in the Matrigel-embedded culture. Monolayer culture accumulated CLF inside the cytoplasm owing to the absence of the lumen whereas rhodamine 123 could hardly be visualised in the monolayer (Fig. 4e). Conversely, the rhodamine 123 was completely transported into the lumen in the Matrigel-embedded culture, even using the same incubation period as in the honeycomb microwell culture.

**Figure 5:**
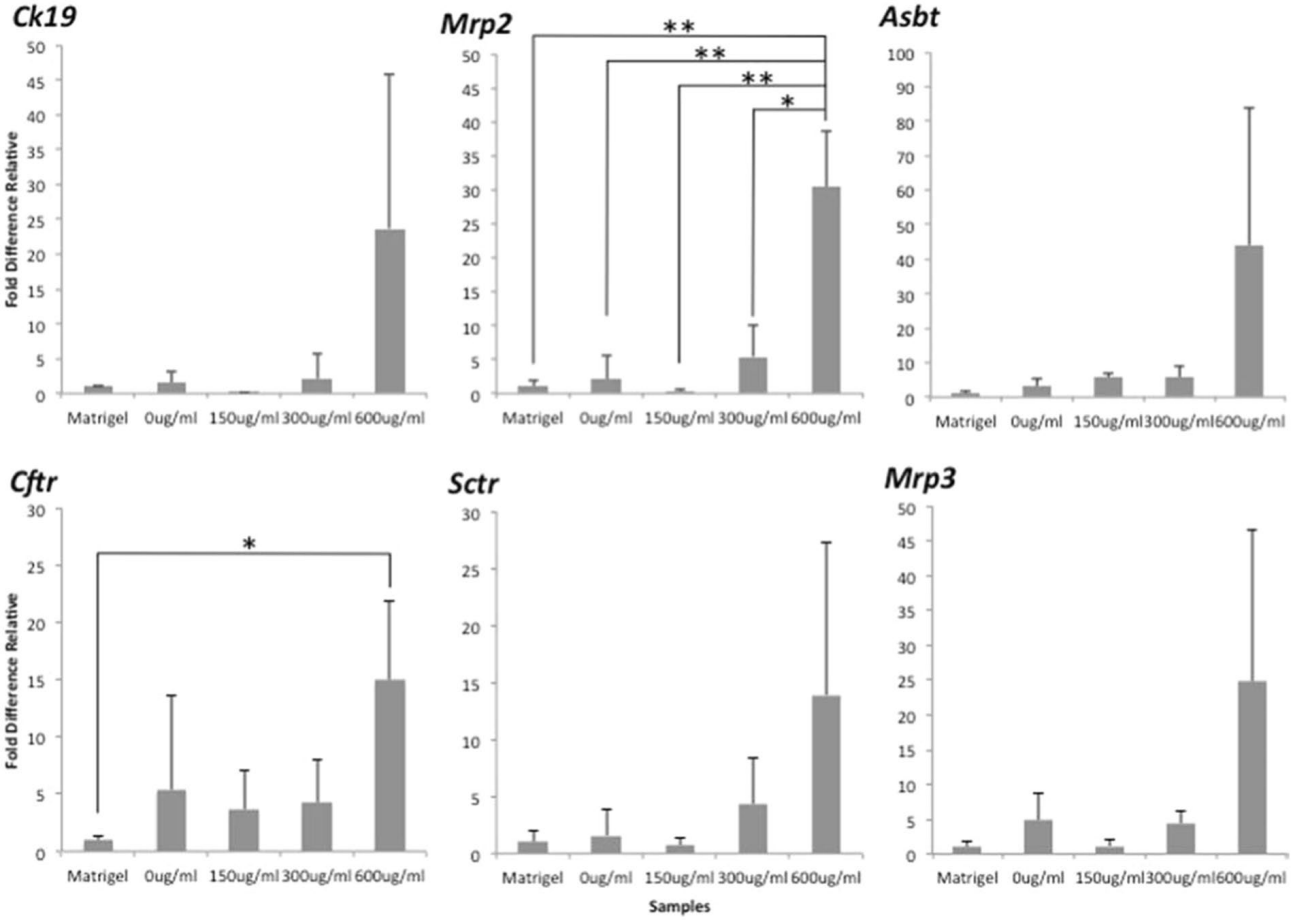
Basic cyst function represented by BEC-related markers as assessed by qRT-PCR at day 3 culture. Quantitative validation is shown of the relative gene expression of the cysts established in 0, 150, 300, and 600 µg/ml concentration with Matrigel-embedded culture as a control. The -actin gene was used as a housekeeping gene. The *Mrp2* and *Cftr* were highly up regulated in the 600 µg/ml concentration (from three independent experiments, n = 3; *p < 0.05; **p < 0.01).

The expression of biliary epithelial-related functions in the various cyst cultures from three independent experiments (n = 3) were measured by quantitative reverse transcription-polymerase chain reaction (qRT-PCR) analyses (Fig. 5) using *Ck19*, *Mrp2*, *Asbt*, *Cftr*, *Sctr*, and *Mrp3* markers and *β*-actin as a housekeeping gene^8,9,26–28^ (Table 2). There was no significant difference in expression of the basic marker of cholangiocytes (*Ck19*), sodium-dependent bile acid transportation (*Asbt*), bidirectional transport of organic ion on the basolateral site (*Mrp3*), and secretin receptor (*Sctr*) in any of the modulated culture conditions. This expression by the cysts in either non-supplemented or low matrigel supplemented (such as 0 and 150 µg/ml) culture condition supposed to be down regulated due to the lack of the presence of ECM. The efflux of bile acid transportation on the apical site can be represented by the activities of multidrug resistance transporter (MRP2) and cAMP regulated Cl^−^ channel (CFTR), the expression of which was significantly up-regulated in 600 µg/ml culture conditions, indicating higher secretion of metabolised fluorescein from and into bile canaliculi. These data did not correspond to the productivity of the cysts exhibited in Fig. 3b, in which the most favourable culture condition was delivered by the 300 µg/ml concentration. In addition, the 600 µg/ml concentration that supported the functional enhancement of the cyst was less reproducible than the 300 µg/ml concentration; such high standard deviation might be consequent to non-homogenous culture condition.

**Table 2:** Primer sequences of specific biliary markers utilised in qRT-PCR assays.

No.	Primer	Gene Target	Sequences (Forward, Reverse)	Size (bp)
1	β-actin	β-Actin, conserved gene	5′-CTGTATTCCCCTCCATCGTG-3′ 5′-GGTGTGGTGCCAGATCTTCT-3′	182
2	CK19	Cytokeratin 19	5′-CCTACCTTGCTCGGATTGAG-3′ 5′-TCACGCTCTGGATCTGTGAC-3′	216
3	MRP2	Multidrug Resistance Transporter 2	5′-CGGTCATCACTATCGCACAC-3′ 5′-GCTAGAGCTCCGTGTGGTTC-3′	180
4	MRP3	Multidrug Resistance Transporter 3	5′-GACAGGCAATGTGAAGCTGA-3′ 5′-GAAAGCTGACAGCATGACCA-3′	244
5	ASBT	Bile Acid Sodium Symporter	5′-GACTCGGGAACGATTGTGAT-3′ 5′-GGTTCAATGATCCAGGCACT-3′	212
6	CFTR	cAMP-regulated chloride channel	5′-GGCAGTACGACTCCCTTCAG-3′ 5′-GAGTTGCTTCCTCAGCATCC-3′	204
7	SCTR	G protein-coupled receptor binds secretin	5′-GGTGGAAGGGCCTCTATCTTC-3′ 5′-GGAAGCGTTGGAGTTGATGT-3′	179

### Cysts represent potential vehicles bile duct establishment *in vitro*

**Figure 6:**
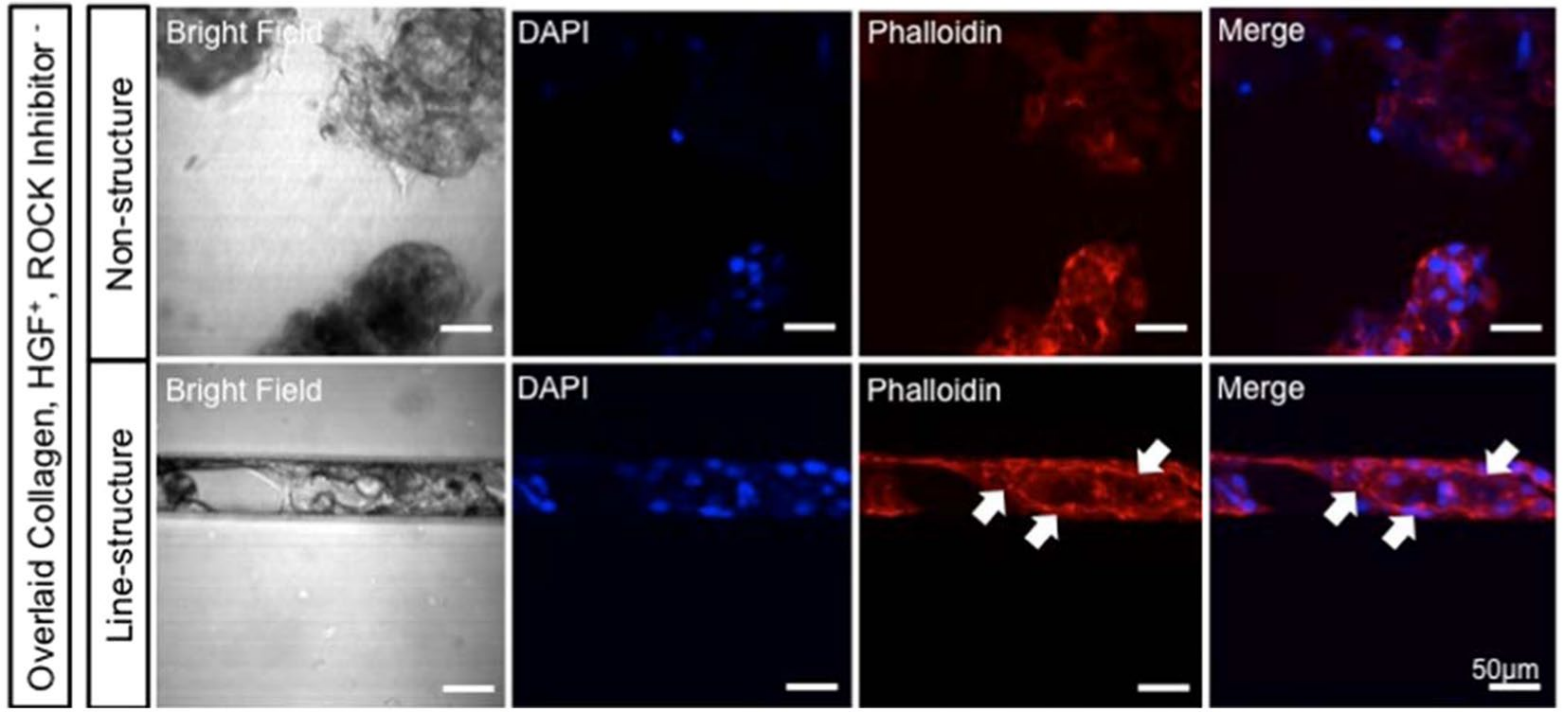
Cysts re-cultured in the 50 µm PDMS-based line microstructure (microcanal), after day 3 culture. Partial bile duct structure was remodelling as shown by actin (phalloidin) and nuclear (DAPI) staining whereas arbitrary structure were observed in the non-structure culture (**a**). Partial creases (white arrows) were found that resemble the tubular structure (**b**). Cysts reformed into a monolayer formation shortly after establishing contact with PDMS surface-coated collagen (scale bar: 50 µm).

During *in vitro* bile duct establishment, we also performed a modulation mimicking the *in vivo* morphogenesis of bile ducts derived from cyst as previously described^14,15^, by employing a PDMS-based line microstructure (microcanal) for further organisation of our cysts. Stable cysts established in ideal culture conditions were harvested and re-cultured in 50, 100, and 200 µm-width size of microcanals (Fig. S3) coated by MPC polymer, employing roughly 1 × 10^5^ cysts/cm^2^. The 50 µm-width size microcanal appeared to serve as a preferable dimension for bile duct culture (data not shown) as it also represents the size-range of a single cyst. The re-cultured cysts on the microcanal were overlaid by collagen type I, along with hepatocyte growth factors (HGF) supplementation and the absence of ROCK inhibitor^29^. Actin (phalloidin) and nuclear (DAPI) staining showed a partial bile duct structure along the microcanal whereas arbitrary structures were observed on the PDMS-non-structure after 3 days culture (Fig. 6a). Partial creases were also found, likely resembling the tubular structure (Fig. 6b). Notably, we also observed that the cysts tended to change their conformation owing to the culture dimension interchange. In particular, the cysts lost their 3D conformation immediately after establishing contact with the 2D surface and reformed into monolayers that were observed as cells adherent on the microcanal.

## Discussion

The bile duct is essential for hepatotoxicology assays owing to its primary role in bile acid collection. However, its *in vitro* establishment is challenging because of its complex structure, as investigated in previous studies through the application of various approaches^6,11–17^. In the present study, we found that modifying the structure of the cyst for recovering bile acid provides a suitable recourse to those approaches. The cyst constitutes the primary structure that has been used to assess the functionality of BECs, in particular during liver cell differentiation studies. It has a 3D structure composition and evinces apicobasal polarity in the laminin-rich ECM, equivalent to the biliary duct in the *in vivo* state^8,9^. Moreover, it is reported as mainly being associated with luminal elongation during biliary duct embryonic morphogenesis^14,15^. Contemplating the essential role of the cysts, we pursued a convenient method to promote efficient BEC-based cyst production *in vitro*. Our method successfully demonstrated an efficient, rapid formation, along with high production of stable and functional cysts through integration of both ideally-dimensioned PDMS-based honeycomb microwells and Matrigel supplementation. Notably, our method simultaneously was able to exhibit that a cyst could be developed from primary adult mouse BECs, how the spatial 3D environment plays a role in cyst development, and the probability of the cyst to further evolve into biliary ducts, mimicking *in vivo* biliary duct morphogenesis.

Our results substantially elaborated that the cyst is initially raised either from single cells or through the aggregation of the BECs. For cysts developed from aggregation, BECs were confined by the honeycomb microwell and subsequently emerged into aggregates. Aggregates comprised of up to six cells were mainly allowed to form and transform their conformation, alternating into cysts only within 24 h, whereas with more than six cells, the structures mostly remained as aggregates. We inferred that the number of cells in a cluster determines the size of the cysts. Cysts established from aggregation were larger than the single cell cysts owing to the number of constituent cells (Fig. 2b,c). We also discovered single-lumen and multi-lumen cysts in the honeycomb microwell. The vast majority of single BECs subsequently only developed into single lumen cysts. However, the evidence indicating the cause of multi-lumen cysts remains unclear as the formed aggregates might further developed into either single-lumen cyst or multi-lumen cysts. Our hypothesis mostly rely upon the composition of aggregates such that big aggregates might remain as aggregates or construct a group of adjacent cysts, which are observed as multi-lumen cysts.

We demonstrated that honeycomb microwell is suitable for mimicking the spatial 3D environment as in conventional Matrigel-embedded culture, in which the dimension plays an essential role for the aggregation and conformation of the cysts. The 46-µm-size appears to be most suitable size as it efficiently and continuously confines clusters of up to six cells. The majority of these clusters aggregated and subsequently conformed into cysts, indicating that this size organised cysts in an acceptable dimension. The primary BECs, which were isolated from mouse using the epithelial adhesion molecule positive (EpCAM^+^) markers, mainly consisted of intrahepatic biliary cells assembling bile ductules (15 µm diameter) or interlobular ducts (15–100 µm diameter)^1^, which nearly equalled the dimension delivered by the 46-size microwells. The aggregates that developed in larger sizes (126 and 326 µm) were mostly failed to develop into cysts. Instead, these sizes might suitably support cyst formation from isolated BECs of hepatic duct origin (diameter ≥ 100 µm)^1^.

The utilisation of optimum Matrigel concentration has several merits over conventional Matrigel-embedded cultures including: curbing overusage of Matrigels, providing a liquid phase-matrix, and enhancing the relevancy of further assays. Preliminary long-term culture (up to 21 days) (Fig. S1) showed that the optimum concentration of Matrigel supplementation was capable of maintaining the formation as well as supporting the subsistence of the cysts similar to that in Matrigel-embedded culture. Otherwise, cysts were unable to persevere after day 5 culture in the less than optimum concentration of Matrigel supplementation. In addition to the spatial support as previously elaborated, the optimum concentration of ECM was positively associated with cyst formation. We perceived that the optimum Matrigel supplementation constantly altered the BECs to form cyst structures, unlike the excessive usage of Matrigel, as evinced by the 300 µg/ml concentration exhibiting significant cyst production compared with that of the Matrigel-embedded culture. Cysts developed at this concentration also formed rapidly, after only 3 days of culture. Matrigel comprises a rich laminin-containing ECM^8–10^, primarily composed of laminin-α1 and laminin-α5^9,17^, which is responsible for BEC polarity determination. The laminin-exposed epithelial membrane is subsequently modified into the basal site facing the matrix^9^, whereas apical sites are developed on the laminin-free membrane facing the lumen site. The liquid phase-matrix provided by Matrigel supplementation enhanced the transportation of fluorescent dyes (as demonstrated with CLF and rhodamine 123 accumulation) as opposed to the Matrigel-embedded culture. The gel phase of Matrigel-embedded culture restrained the transportation of the dye, resulting in faded fluorescence observed in the lumen and accumulation of both dyes within the cells (Fig. 4e). The liquid phase-matrix also allowed more convenient cyst harvesting by eliminating the usage of cell recovery solutions. Conversely, in the case of Matrigel-embedded culture, the enzymatic process delivered by the recovery solution leaves the undigested Matrigel residue, which might either interfere with downstream analyses or damage the cyst structure upon an excess incubation. Thus, our methods successfully diminish these drawbacks.

Our study suggests that spatial modulation or presence of a matrix might not independently represent the main factor of cyst functional enhancement per se; instead, our results elaborated that the well dimension and ECM were synergistic for derived-biliary tissue development. Specifically, the combination of spatial control and adequate ECM concentration was critical for mobility of the cells to form an organoid (cyst) structure. The confinement afforded by the microwell and liquid-phase culture environment provided by ECM supplementation permitted the cell mobility to form aggregates and subsequent cysts as reflected by the rapid formation and maturation of the cysts in only a 3-day culture. This facilitation was the strongest 300 µg/ml Matrigel concentration as demonstrated by the highest number of cysts, whereas the expression of basic BEC, apical, and basal polarity markers, along with bile acid efflux towards and outward of the structure, were exceeded by the values obtained at the 600 µg/ml concentration. This also elaborates that cysts established in honeycomb microwell with no or low supplementation maintain favourable expression of BEC markers compare to that in the Matrigel-embedded culture owing to the faster 3D conformation, although the expression decreased significantly after day 5 of culture as a result of ECM deficiency (wherein the majority of cysts had burst). In the Matrigel-embedded culture, we assumed that the gel-phase culture environment prevented the mobility of the cells required to form aggregates, so that the cysts observed in this culture were mainly developed from single cells and appeared as semi-cysts. In other words, the productivity of cysts in the Matrigel-embedded culture was less favourable than that upon optimum supplementation at the same day-age culture. We also considered that the Matrigel residue might interfere with the RNA assays in qRT-PCR analyses, with the excess in the Matrigel-embedded culture thus limiting the expression of the gene markers.

The cyst we obtained was distinct in cell proliferation from the typical cyst-structure reported previously, which consists of many cells and continuously proliferates throughout its growth^8,9,25^. The difference in the cyst formation may be caused depending on the staring cell type; our structure was established from primary isolated EpCAM^+^ cells, which include mature BECs as well as adult hepatic progenitor cells whereas the recorded cysts prevalently arose from developmental progenitor cells, derived from mouse fetal hepatoblasts or human iPSCs^9,25^. Regardless, both types evinced equal function with respect to bile acid accumulation. Notably, our method is simple to perform and requires less biliary-specific inducer than that necessary for differentiation. It is thus favourable for assays related to BEC function including cyst-like structures for iPSC-to-BEC differentiation, bile acid accumulation, and for further experiments that require high numbers of harvested cysts.

In *in vitro* bile duct establishment, fluorescence staining confirmed that cysts cultured in the microcanal overlaid with collagen type I gel showed a probability of bile duct formation. Our microcanal satisfyingly governed the motility and the structure of the tubular structure during luminal elongation. We believe that the HGF supplementation, removal of ROCK inhibitor, and collagen type I overlay are responsible for the partial crease formation of the BEC cysts over the microcanal. Based on a previous study, the presence of HGF likely triggered luminal elongation of the MDCK cysts whereas the absence of the ROCK inhibitor aborted the segmentation of the tubular structure^29^. In particular, the ROCK inhibitor activates the p114RhoGEF–ROCK1–myosin-IIA pathway responsible for the limitation of cell motility and induces segmentation of the tubular structure. Moreover, collagen type I is also suitable for cell proliferation and thus provides an increased possibility for the partial creases to extend and fully sheath over the microcanal. We found that Matrigel is less desirable for luminal elongation whereas it more likely suitable for cell differentiation culture (Fig. S4). Considering the culture dimension interchange, we assumed that the 2D surface mostly altered the polarity of the cysts, thus triggering monolayer formation, although the mechanism remains unclear. Eventually, our model has potential implications regarding the disease models, particularly for intrahepatic cholestasis testing. For example, by disturbing the bile acid-related transporters on the cysts, polarity changes can be applied in evaluating drug transport^30^. Our method also elaborated that a continuous bile duct structure could feasibly be established *in vitro*, which would be is desirable for bile duct-engineering studies. Bile duct establishment that includes the tubulogenesis involves complex signalling including cell fate determination by Notch2 and Jagged 1^8,10,13^ provided by neighbouring tissues. Thus, cyst modulation alone is entirely inadequate to initiate bile duct establishment. Going forward, we are working on the development of a continuous bile duct culture employing the combination of these signaling pathways. Recent studies have focused on developing the most efficient method to recover bile acid using i*n vitro* culture. We believe that the establishment of a continuous bile duct establishment *in vitro* would provide a suitable method for cell-based bile acid recovery and relevant liver-tissue engineering.

## Materials and Methods

### Fabrication of PDMS-based honeycomb microwells

A silicon mould was fabricated using SU-8-based negative photolithography on a silicon wafer as previously described^20,21,31^ with the various diameters: 46, 76, 126, and 326 µm for honeycomb microwells^20^ (Fig. 1a); 50, 100, and 200-µm-size width for line-microcanal (Fig. S3); followed by CHF_3_ surface coating^20^. Subsequently, PDMS honeycomb microwells and line-microstructures were generated using the replica moulds and MPC co-polymer coating was performed after the O_2_ plasma treatment^20,23,24^ (Fig. 1b). The PDMS sheets were then independently inserted onto PDMS-bottom 96-well plates^20,22,32^ (Fig. 1c,d) aseptically and subsequently surface washed by phosphate buffer saline (PBS) to remove debris.

### Primary BEC isolation

The mouse BECs primary cells were isolated from 8–12 weeks old male C57BL/6 mice (CLEA Japan, Tokyo, Japan) employing a modified two step standard collagenase liver perfusion protocol^3,5,6,33^ followed by an EpCAM^+^ cell sorting protocol as described previously^34,35^. Briefly, the non-parenchymal cell suspension was incubated with anti-Fc receptor (FcR) antibody for FcR blocking, followed by incubation with anti-EpCAM antibody conjugated with fluorescence isothiocyanate (FITC). After the enrichment of EpCAM^+^ cells by magnetic activated cell sorting (MACS) using anti-FITC microbeads (Miltenyi Biotec), the EpCAM^+^ cell fraction was further purified by fluorescence-activated cell sorting (FACS) with Moflo XDP (Beckmann courter, Brea, CA). The sorted EpCAM^+^ cells were cultured with William’s E medium (Gibco) supplemented with a final concentration of 10% fetal bovine serum (Bioserra), 10 mM nicotinamide (Sigma), 2 mM L-glutamine (Gibco), 0.2 mM ascorbic acid (Wako), 20 mM Hepes (Sigma), 1 mM Na-pyruvate (Gibco), 0.15% sodium hydrogen carbonate (NaHCO_3_), 14 mM L-glucose (Sigma), and 50 μg/mL of gentamycin (Wako). The additional supplementation mixture for the above-mentioned medium: 1 × insulin inhibitor solution (Gibco), 1 × 10^−7^ dexamethasone, 1 × ROCK inhibitor/Y27632 (Sigma), 10 ng/ml mouse HGF, and 10 ng/ml mouse epidermal growth factor was added immediately prior to use. The animal experiments were conducted in accordance with institutional procedures and approved by the Animal Care and Use Committee of the Institute of Molecular and Cellular Biosciences, The University of Tokyo (approval number 2706), following the guidelines of the Japanese Ministry of Education.

### Cyst formation

To generate the cysts structure, BECs were seeded on the honeycomb microwell (on PDMS-bottom 96-wells O_2_ permeable plates) (Fig. 1e) and centrifuged at 30 g for 5 min with minimum acceleration and deceleration to retain the BECs inside the microwell. Cultures were incubated at 37 °C in 5% CO_2_. A series of Matrigel (Gibco) supplementations were conducted 1 day after inoculation (Fig. 1f) to determine the optimum condition for cyst formation: 0, 150, 300, 600 μg/ml and 1, 3, 6 mg/ml, and original Matrigel concentration (9.2 mg/ml). Culture medium was changed every other day with Matrigel-supplemented culture medium. To investigate the aggregation and conformation of BECs into cysts, a real-time recording (JuliBr Live Cells Movie Analyzer) was performed within 24 h with 1 h intervals (Fig. 2d).

### Bile duct establishment *in vitro*

Cysts were harvested from the optimum cyst culture and re-inoculated in the PDMS-line microcanal (inserted into PDMS-bottom 96-well plates)^20,23,24^ followed by centrifugation at 30 g for 5 min with minimum acceleration and deceleration. Primary BEC culture medium was used without ROCK inhibitor/Y27632 supplementation. Collagen type I gel (Biosera) was overlaid 1 day after inoculation.

### Cytomorphology remodelling

Phalloidin-conjugated Alexa 647 (Santa Cruz) staining was conducted to reconstruct the actin filament for cytological morphology. After fixation by 4% paraformaldehyde at 4 °C overnight, the cyst was then stained by 1 × phalloidin (dissolved in PBS with 1% bovine serum albumin) for 3 h followed by DAPI (Dojindo), 1/1000 in PBS for 5 min for nuclear staining. The structure was examined by confocal microscopy using Alexa Fluor 647 (far red) for phalloidin and DAPI (blue) emission spectra for DAPI. The 3D-stacked image series was later analysed using ImageJ software.

### Bile transporter activities of the cyst

The bile acid conjugate CLF has been used for visualisation of bile acid transportation across liver tissue^3,5,6,25^, in particular as regulated by MRP2 whereas the activities of MDR1 was assessed by rhodamine 123 efflux^8,9,19^. Independently, cysts were washed and incubated separately with 5 μM CLF (BD, Japan) and 100 μM rhodamine 123 (BD) in the free serum-culture medium, at 37 °C within 30 min. Subsequently, cysts were rinsed twice with fresh culture medium. The fluorescence dye accumulation was then recorded using a confocal microscope (Olympus, Japan) employing FITC (495–519 nm) for CLF and rhodamine green (502–527 nm) emission spectra.

### Qualitative assays of genes expression

Cyst samples were dispersed in 500 μl Trizol reagent (Invitrogen) by pipetting. Chloroform (Wako), as much as 100 μl was added into the samples, mixed, incubated at room temperature for 5 min, and centrifuged at 12000 g for 15 min. The transparent aqueous phase was separated, mixed with 250 μl isopropyl alcohol (Wako), incubated for 15 min at RT, and centrifuged at 12000 g for 15 min. Pellet on the bottom of the microtube was washed by 1 ml EtOH, mixed, and centrifuged at 7500 g for 5 min. After removed the EtOH, 10 μl RNAase free water was added to the pellet and mixed. RNA samples with concentration at least 100 μg/ml were used to obtain cDNA. An RT reaction was obtained using the Tra Ace qPCR RT Master Mix with gDNA Remover (Toyobo, FSQ-301) protocols employing these phases: 37 °C, 15 min; 50 °C, 5 min; 98 °C, 5 min; and 4 °C, hold. Subsequently, we qualitatively assessed the expression of biliary cell-related function by qRT-PCR using several specific biliary markers followed by cDNA amplification (Thunderbird Sybr qPCR mix) using a real-time PCR (StepOnePlus) system. We analysed six markers (Eurofins) (Table 2) to assess gene expression of the cysts with *β*-actin serving as a housekeeping gene and employing this cycling condition: pre-denaturation at 95 °C for 60 sec, denaturation at 95 °C for 15 sec, and extension (40 cycles) at 60 °C for 60 sec.

### Statistical analyses

Results were represented as the means ± s.d. Relative gene expression was calculated using comparative Ct from several independent experiments. All data were analysed with a two way student t-test to determine the significances between samples with p < 0.05 and p < 0.01 considered statistically significant and p < 0.001 as highly significant.

## Electronic supplementary material

Supporting Information

## References

[1] Han Y (2013). Recent advances in the morphological and functional heterogeneity of the biliary epithelium. Exp Biol Med (Maywood).

[2] Fu D, Wakabayashi Y, Ido Y, Lippincott-Schwartz J, Arias IM (2010). Regulation of bile canalicular network formation and maintenance by AMP-activated protein kinase and LKB1. Journal of Cell Science.

[3] Xiao W, Perry G, Komori K, Sakai Y (2015). New physiologically-relevant liver tissue model based on hierarchically cocultured primary rat hepatocytes with liver endothelial cells. Integrative Biology.

[4] Bale SS, Geerts S, Jindal R, Yarmush ML (2016). Isolation and co-culture of rat parenchymal and non-parenchymal liver cells to evaluate cellular interactions and response. Scientific Reports.

[5] Matsui H, Takeuchi S, Osada T, Fujii T, Sakai Y (2012). Enhanced bile canaliculi formation enabling direct recovery of biliary metabolites of hepatocytes in 3D collagen gel microcavities. Lab Chips.

[6] Katsuda T, Kojima N, Ochiya T, Sakai Y (2013). Biliary epithelial cells play an essential role in the reconstruction of hepatic tissue with a functional bile ductular network. Tissue Engineering.

[7] Bell CC (2016). Characterization of primary human hepatocytes spheroids as a model system for drug-induced liver injury, liver function, and disease. Scientific Reports.

[8] Ogawa M (2015). Directed differentiation of cholangiocytes from human pluripotent stem cells. Nature Biotechnology.

[9] Tanimizu N, Miyajima A, Mostov KE (2007). Liver progenitor cells develop cholangiocyte-type epithelial polarity in three-dimensional culture. Molecular Biology of the Cell.

[10] Matsumoto S (2014). A combination of Wnt and growth factor signaling induces Arl4c expression to form epithelial tubular structures. The EMBO Journal.

[11] Lemaigre FP (2003). Development of biliary tract. Mechanism of development.

[12] Si-Tayeb K, Lemaigre FP, Duncan SA (2010). Organogenesis and development of the liver. Developmental Cell.

[13] Antoniou A (2009). Intrahepatic Bile Ducts Develop According to a New Mode of Tubulogenesis Regulated by the Transcription Factor SOX9. Ganstroenterology.

[14] Vestentoft PS (2011). Three-dimensional reconstructions of intrahepatic bile duct tubulogenesis in human liver. BMC developmental biology.

[15] Takashima Y, Terada M, Kawabata M, Suzuki A (2015). Dynamic three-dimensional morphogenesis of intrahepatic bile ducts in mouse liver development. Hepatology.

[16] Zegers MMP, O’Brien LE, Yu W, Datta A, Mostov KE (2013). Epithelial polarity and tubulogenesis *in vitro*. TRENDS in cell biology.

[17] Tanimizu N, Mitaka T (2016). Morphogenesis of liver epithelial tissue. Hepatology Research :Review Article.

[18] Anzai K (2016). Feotal hepatic progenitor cells assume a cholangiocytic cell phenotype during two-dimensional pre-culture. Scientific reports.

[19] Sampaziotis F (2015). cholangiocytes derived from human induced pluripotent stem cells for disease modeling and drug validation. Nature Biotechnology.

[20] Shinohara M (2013). Combination of microwell structures and direct oxygenation enables efficient and size-regulated aggregate formation of an insulin secreting pancreatic *β*-cell line. Biotechnol. Prog..

[21] Goral VN, Au SH, Faris RA, Yuen PK (2015). Methods for advanced hepatocyte cell culture microwells utilizing air bubbles. Lab Chip.

[22] Hamon M, Hanada S, Fujii T, Sakai Y (2012). Direct Oxygen Supply With Polydimethylsiloxane (PDMS) Membranes Induces a Spontaneous Organization of Thick Heterogeneous Liver Tissues From Rat Fetal Liver Cells *In Vitro*. Cell Transplantation.

[23] Isihara K, Ishikawa E, Iwasaki Y, Nakabayashi N (1999). Inhibition fibroblast cell adhesion on substrate by coating with 2-methacryloyloxyethyl phosphorylcholine polymer. J Biomater. Sci. Polym..

[24] Ukawa M (2010). 2-Methacryloyloxyethyl phosphorylcholine polymer (MPC)-coating improves the transfection activity of GALA-modified lipid nanoparticles by assisting the cellular uptake and intracellular dissociation of plasmid DNA in primary hepatocytes. Biomaterials.

[25] Dianat N (2014). Generation of Functional Cholangiocyte-Like Cells From Human Pluripotent Stem Cells and HepaRG Cells. Hepatology.

[26] Hashimoto W (2008). Ductular network formation by rat biliary epithelial cells in the dynamic culture with collagen gel and dimethylsulfoxide stimulation. The American Journal of Pathology.

[27] Tabibian JH, Masyuk AI, Masyuk TV, O’Hara SP, LaRusso NF (2013). Physiology of cholangiocytes. Comparative Physiology.

[28] Froster S, Thumser AE, Hood SR, Plant N (2012). Characterization of rhodamine-123 as a tracer dye for use in *in vitro* drug transport assays. PLoS One.

[29] Kim M, Shewann AM, Ewald AJ, Werb Z, Mostov KE (2015). p11RhoGEF governs cell motility and lumen formation during tubulogenesis through a ROCK-myosin-II-pathway. The company of Biologists.

[30] Burbank MG (2016). Eraly alterations of bile canaliculi dynamics and the rho kinase/myosin light chain kinase pathway are characteristics of drug-induced intrahepatic cholestasis. Drug Metabolism and Disposition.

[31] Provin C, Takano K, Sakai Y, Fujii T, Shirakasi R (2008). A method for the design of 3D scaffolds for high-density cell attachment and determination of optimum perfusion culture conditions. Journal of biomechanics.

[32] Evenou F, Hamon M, Fujii T, Takeuchi S, Sakai Y (2011). Gas permeable membranes and co-culture with fibroblasts enable high-density hepatocyte culture as multilayered liver tissue. Biotechnol. Prog..

[33] Sargent NSE, Oestreichert M, Haidvoglt H, Madnickt HM, Burger MM (1988). Growth regulation of cancer metastases by their host organ. Proc. Natl. Acad. Sci. USA..

[34] Okabe M (2009). Potential hepatic stem cells reside in EpCAM^+^ cells of normal and injured mouse liver. Development.

[35] Tanaka, M. *et al*. Mouse hepatoblasts at distinct developmental stages are characterized by expression of EpCAM and DLK1: Drastic change of EpCAM expression during liver development. *Mech Dev*. **126**, 665–676, 10.1016/j.mod.2009.06.939 (2009). 10.1016/j.mod.2009.06.939 19527784

