# Supplementary material for: Efficient functional cyst formation of biliary epithelial cells using microwells for potential bile duct organisation in vitro
Source: Sci Rep. 2018 Jul 23;8:11086. doi: 10.1038/s41598-018-29464-w (PMC6056467; doi:10.1038/s41598-018-29464-w)
Supplement: Supplementary file 1 — Supporting Information [file 41598_2018_29464_MOESM1_ESM.pdf]

Efficient functional cyst formation of biliary epithelial cells using microwells for potential bile duct organization *in vitro*

*Astia RIZKI-SAFITRI[1], Marie SHINOHARA[1], Yasushi MIURA[2,5], Mathieu DANOY[3,4], Minoru TANAKA[5,6], Atsushi MIYAJIMA[5], and Yasuyuki SAKAI [1,7,8]

[1]Center for International Research on Integrative Biomedical Systems (CIBiS), Institute of Industrial Science (IIS), The University of Tokyo, Japan
[2]Department of Life Science and Medical Bio-Science, School of Advanced Science and Engineering, Waseda University, Japan
[3]LIMMS/CNRS UMI2820, Institute of Industrial Science (IIS), The University of Tokyo, Japan
[4]Institut d'Electronique, de Microélectronique et de Nanotechnologies (IEMN), Université Lille, Lille, France.
[5]Laboratory of Cell Growth and Differentiation, Institute of Molecular and Cellular Bioscience, The University of Tokyo, Japan,
[6]National center for global medicine (NCGM), Tokyo, Japan,
[7]Department of Chemical System Engineering, Graduate School of Engineering, The University of Tokyo, Japan,
[8]Max Planck-The University of Tokyo, Center for Integrative Inflammology, The University of Tokyo, Japan.

*) Corresponding author

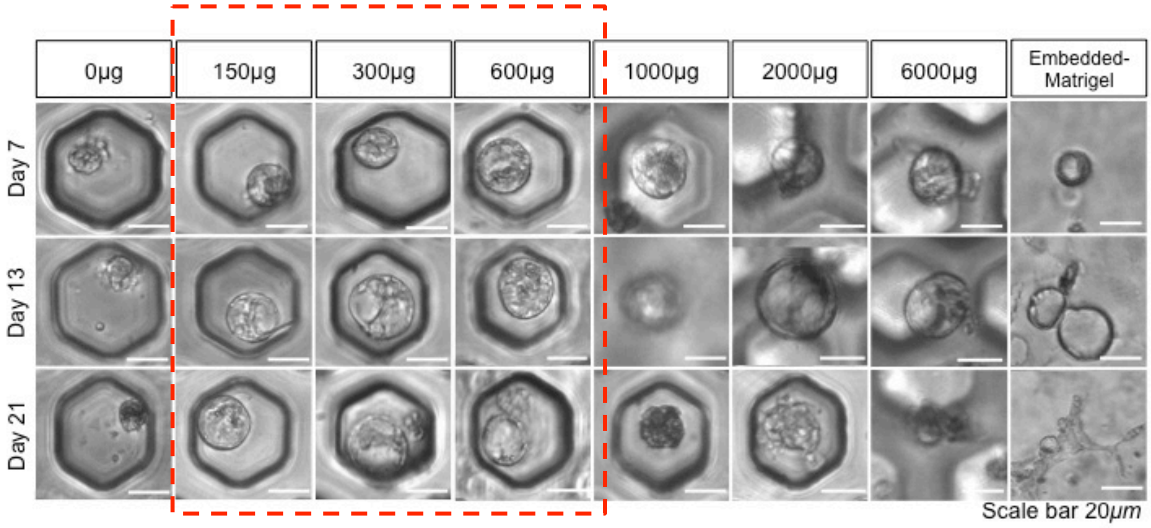

Supplement 1. Cyst morphology in the various concentrations of Matrigel were recorded within 25 days and showed that the most cysts were burst after day 13. High concentration of Matrigel (more than 600 µg/ml) allowed cyst migration outside the microwells and promoted the fusion of several cysts into larger aggregates. Meanwhile, low concentrations (less than 150 µg/ml) could not maintain the cysts viability more than 5 days. Cysts in the 150, 300, and 600 µg/ml Matrigel concentration (red dashed-square) exhibited morphology similar to Matrigel-embedded culture (scale bar 20: µm).

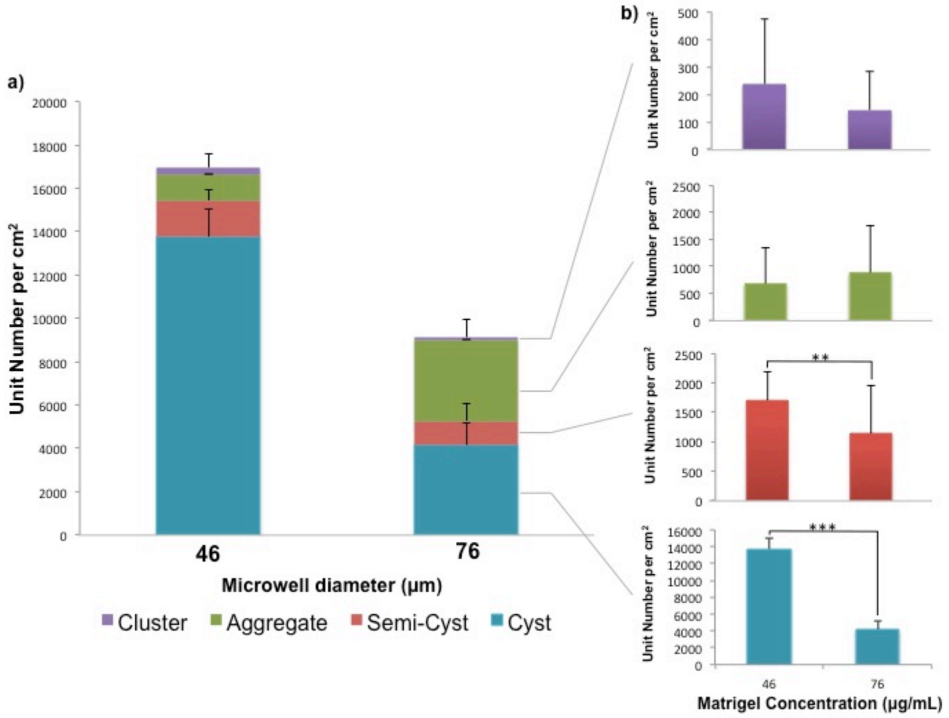

Supplement 2. Comparison of average numbers of four different types of structures per well in 46- and 76-µm-size honeycomb microwells in optimum Matrigel supplementation (300µg/ml) after 3 days culture. Data were collected from 30 images from 3 independent experiments (n = 3) with standard deviation. Structures established in 46-µm-size were significantly larger than the 76-µm-size (**a**) owing to the large number of microwells in the 46-µm-size (**b**), ($^{*}p < 0.05$; $^{**}p < 0.01$; $^{***}p < 0.001$).

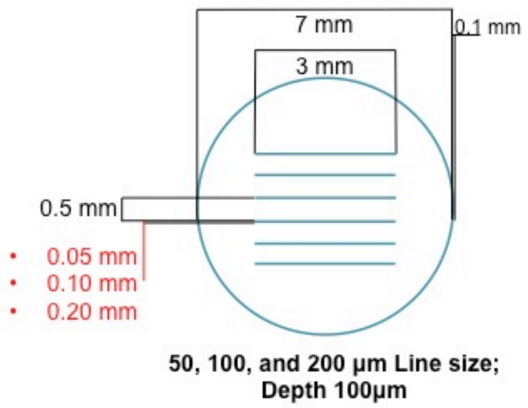

Supplement 3. PDMS-line microstructure (microcanal) design for photomask: 50, 100, and 200-µm-size with 100 µm depth.

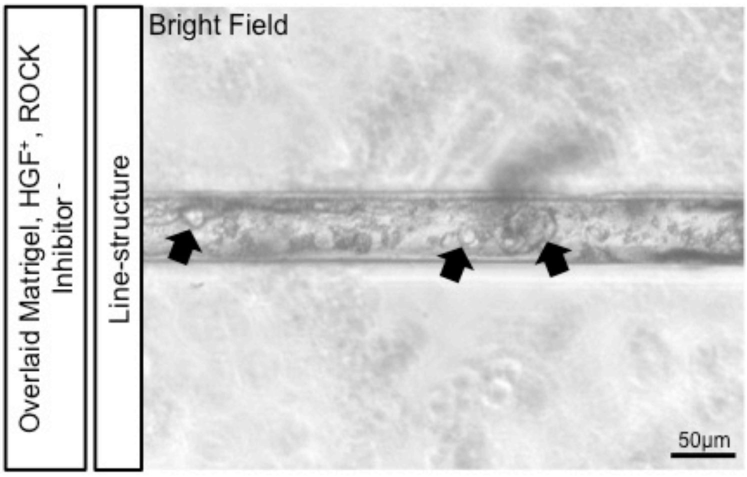

Supplement 4. Cysts were re-cultured in the 50 µm-size PDMS-based line microstructure (microcanal), overlaid by Matrigel after 3 days culture. Most of the cysts in the microcanal were remained as independent cyst (black arrow) without connected to another cyst as observed using bright field microscope (scale bar 50: µm).
